# Could DNA uptake be a side effect of bacterial adhesion and twitching motility?

**DOI:** 10.1007/s00203-013-0870-1

**Published:** 2013-02-05

**Authors:** M. Bakkali

**Affiliations:** Departamento de Genética, Facultad de Ciencias, Universidad de Granada, Fuentenueva S/N, 18071 Granada, Spain

**Keywords:** Competence, DNA uptake, Transformation, Horizontal gene transfer, Type IV pilus, Twitching motility, Social gliding, Swarming, Bacteria

## Abstract

DNA acquisition promotes the spread of resistance to antibiotics and virulence among bacteria. It is also linked to several natural phenomena including recombination, genome dynamics, adaptation and speciation. Horizontal DNA transfer between bacteria occurs via conjugation, transduction or competence for natural transformation by DNA uptake. Among these, competence is the only mechanism of transformation initiated and entirely controlled by the chromosome of the recipient bacteria. While the molecular mechanisms allowing the uptake of extracellular DNA are increasingly characterized, the function of competence for natural transformation by DNA uptake, the selective advantage maintaining it and the reasons why bacteria take up DNA in the first place are still debated. In this synthesis, I review some of the literature and discuss the four hypotheses on how and why do bacteria take up DNA. I argue that DNA uptake by bacteria is an accidental by-product of bacterial adhesion and twitching motility. Adhesion and motility are generally increased in stressful conditions, which may explain why bacteria increase DNA uptake in these conditions. In addition to its fundamental scientific relevance, the new hypothesis suggested here has significant clinical implications and finds further support from the fact that antibiotics sometimes fail to eliminate the targeted bacterium while inevitably causing stress to others. The widespread misuse of antibiotics may thus not only be selecting for resistant strains, but may also be causing bacteria to take up more DNA with the consequent increase in the chances of acquiring drug resistance and virulence—a scenario in full concordance with the previously reported induction of competence genes by antibiotics in *Streptococcus pneumoniae* and *Legionella pneumophila*.

Acquisition of new genetic material by bacteria occurs by transduction, conjugation or competence for natural transformation by uptake of extracellular DNA (hereafter referred to simply as competence). These mechanisms of horizontal gene transfer (HGT) are sources of genetic diversity in the otherwise mostly clonal world of bacteria and can even interfere with speciation by blurring species boundaries [see (Fraser et al. 2007)]. HGT is central to the spread of virulence and resistance to antibiotics among human and livestock pathogens (Vazquez et al. 2001; Courvalin 2005; Sabia et al. 2005; Bartlett 2006; Diep et al. 2006; Messi et al. 2006) (e.g. Methicillin-resistant *Staphylococcus aureus*, Vancomycin-resistant enterococci, including *Clostridium difficile*, and more virulent/resistant *Neisseria meningitides* and *Neisseria gonorrhoeae*). Indeed, the relatively recent deaths caused by a chimeric, virulent and multidrug-resistant *Escherichia coli* strain in Europe are just a small reminder of the serious implications of such natural phenomenon. Neither conjugation nor transduction are initiated by the recipient cell, and HGT through those DNA transfer mechanisms is a side effect of plasmid transmission (which are symbiotic, accessory or selfish elements) and infection by bacteriophages (which are parasites). Contrarily, the chromosome of the recipient cell initiates and entirely controls competence, making this the only mechanism through which acquisition of foreign DNA molecules is not perceived as side effect (Redfield 2001).

Discovered by Frederick Griffith (Griffith 1928) and best defined by David Dubnau as ‘a genetically programmed physiological state permitting the efficient uptake of macromolecular DNA’ (Dubnau 1999), competence is a complex heterogeneous phenomenon. Indeed (1) most bacteria were never reported as competent, whereas some bacteria are known to develop such state. (2) Competence can be constitutive [e.g. *Neisseria meningitidis* and *N. gonorrhoeae* (Catlin 1960; Biswas et al. 1977)] or occasional [e.g. *Bacillus subtilis*, *Haemophilus influenzae* and *Streptococcus pneumoniae* (Singh and Pitale 1968; Herriott et al. 1970; Havarstein et al. 1995)]. (3) Its inducers are species specific [e.g. temperature and media alterations (Herriott et al. 1970; Ranhand 1976; Lopez et al. 1983; Auzat et al. 1999; MacFadyen et al. 2001)]. (4) Depending on the species, the fraction of competent cells varies from ≤25 % [e.g. *Pseudomonas stutzeri*, *Streptomyces virginiae*, *Streptomyces kasugaensis*, *B. subtilis* and *Acinetobacter calcoaceticus* (Roelants et al. 1976; Smith et al. 1981; Lorenz et al. 1992; Palmen et al. 1993)], to ~100 % [e.g. *Azotobacter vinelandii*, *H. influenzae* and *S. pneumoniae* (Smith et al. 1981; Glick et al. 1985)]. (5) Most competent species take up DNA indiscriminately, but some ‘prefer’ con-specific DNA [see (Lorenz and Wackernagel 1994; Dubnau 1999)]. (6) In competent Pasteurellaceae and Neisseriaceae species, the ‘preference’ toward the bacterium’s own species DNA requires that the incoming DNA contains short specific sequences called DNA uptake enhancing sequences (DUES) (Sisco and Smith 1979; Graves et al. 1982; Bakkali 2007). In *A. vinelandii*, *Campylobacter coli* and *P. stutzeri*, however, the preferential uptake of con-specific DNA requires no DUES (Doran and Page 1983; Wang and Taylor 1990).

Except from few differences relating to the presence/absence of an outer membrane, development of competence is similar between Gram-positive and Gram-negative bacteria and consists of (1) DNA binding, (2) exonuclease degradation of its 3′ strand simultaneous to (3) the interiorization of the 5′ strand, and finally, (4) digestion of the incoming single stranded DNA in the cytoplasm or, occasionally, its recombination with the cell’s chromosome (Lorenz and Wackernagel 1994; Dubnau 1999; Chen and Dubnau 2003; Chen et al. 2005). Research on the genetic control of the competent state shows that it involves a complex network of genes [see (Dubnau 1999; Chen and Dubnau 2003; Chen et al. 2005)] controlling a variety of processes including oxidative, nucleic acids and sugar metabolism (Macfadyen et al. 1996; Auzat et al. 1999; MacFadyen et al. 2001), DNA recombination and repair (Raymond-Denise and Guillen 1992; Kruger et al. 1997; Mortier-Barriere et al. 1998), quorum sensing (Li et al. 2002), heat shock (Turgay et al. 1997) and secretion systems (Chen and Dubnau 2003; Averhoff 2004).

In contrast with the scientific advances and consensus on the nature of the molecular basis and series of events that take place during competence, the origin, reasons of being, maintenance and evolution of this phenomenon are still subjects of ongoing debate. The phylogenetic origin and distribution of competence are hitherto undefined. Lorenz and Wackernagel reported ~40 naturally competent species (Lorenz and Wackernagel 1994), and since then, ~20 more were added, and this number will certainly keep increasing. Competence could therefore be more common than we currently know. Its scattered distribution across taxa ostensibly suggests independent evolution. However, scattering could result from bias in research and from the small number of species reported as competent compared to the number of phylogenetic branches. In fact, the likelihood of such a complex phenomenon independently evolving in several lineages is negligible. Competence is probably an ancestral feature of at least most lineages—a possibility supported by findings in (Redfield et al. 2006). Species not known as competent could therefore be competent under unknown conditions or may have lost this ability. For instance, *Pseudomonas fluorescens* and *Agrobacterium tumefaciens* seem competent in nature but not under laboratory conditions (Demaneche et al. 2001), whereas *Pasteurella multocida* was never reported as competent although it belongs to a family of competent species and has the genes involved in competence and the DUES ‘preferred’ by *H. influenzae* (Bakkali et al. 2004).

To explain the evolution and maintenance of competence, four hypotheses were suggested. Each of these was the result of neat logic and great intellectual formulations and was supported by elegantly designed experiments (see references herein). Nonetheless, the matter is still subject of controversy as none of these hypotheses can explain all the cases of competent species. A new hypothesis is therefore needed to provide a unifying explanation and account for the weaknesses on which I will focus my following discussion of each of the four existing hypotheses.

*Competence for chromosome repair* (Michod et al. 1988) relies on the up-regulation of DNA repair genes in competent *B. subtilis* (Love et al. 1985) and the increase in its transformation and survival when cells are UV treated before adding DNA to the medium, but not after (Michod et al. 1988). However, it is argued that DNA repair cannot explain the increase in survival, that extracellular DNA is often damaged and useless for chromosome repair, and that DNA damaging agents do not induce competence neither in *H. influenzae* nor in *B. subtilis* (Mongold 1992; Redfield 1993a). Yet, there is a plethora of situations where most extracellular DNA will not be damaged (e.g. in host tissues or in soil). Nonetheless, to me, the repair hypothesis has at least three major weaknesses: First, most of the DNA taken up is eliminated/degraded (Lorenz and Wackernagel 1994; Dubnau 1999; Chen and Dubnau 2003; Chen et al. 2005). Although no energy cost is too high if the final outcome is survival, eliminating one DNA strand at the cell surface means spending energy and a 50 % reduction in the chances of successful chromosome repair. Subsequent cytoplasm-localized degradation of the DNA means further energy cost and reduction in the repair material that DNA might be—why should bacteria ‘waste’ that repair material as well the energy required for degrading it if what they want is chromosome repair? It is true that *S. pneumoniae*, *L. pneumophila* and probably other competent bacteria seem to lack the standard SOS DNA repair system, so that any contribution to the repair system might be selected for, many competent bacteria already possess the standard DNA repair mechanisms and, in principle, would not need competence for repair. Second, it is DNA uptake and its entry into the cytoplasm what induces the DNA repair machinery and not the reverse—up-regulation of the DNA repair genes seems to be an SOS response to the presence of single stranded DNA inside the cell. Third, and probably more importantly, uptake of DNA that corresponds to the damaged chromosomal region is so unlikely for it to explain the evolution of competence, especially since most competent bacteria take up DNA indiscriminately and not only from their own species. One may argue that restriction–modification and cytoplasmic nucleases would eliminate the non-homologous DNA. The benefit of competence as repair mechanism would then depend on the prevalence of the homologous DNA compared to the non-homologous one. In high population densities, one could expect the DNA from the same species to be frequent but, in a non-homogenous medium and in hosts, most of the DNA would come from other species (for instance, most of the DNA in human lung would be human and not from the pathogen). Furthermore, even if the homologous were frequent enough, it still must contain the damaged region of the chromosome that the bacterium seeks to repair and this would depend on the length of the DNA fragment. It is true that bacteria can take up large DNA fragments but, outside the cell, DNA tends to be degraded and fragmented due to the action of the many agents, chemical and physical, as well as to the different enzymes secreted by other living beings. Indeed, even if the DNA taken up was always from the own species and as big as 20 kb and that the bacterial genome were small as 1 Mb, in 98 % of the times, the competent bacterium will take up DNA that would be useless for repairing its chromosome. In a way, one may make the analogy between taking up random DNA fragments for chromosome repair and buying random mechanical pieces with the hope that one might repair a damaged car engine.

*Competence for recombination* is a widely accepted idea recognizing competence as a mechanism for genetic diversification comparable to eukaryotic sex [see (Narra and Ochman 2006)]. DUESs could therefore be mate recognition tags (Redfield 1991) preventing interspecific HGT [e.g. DUESs would prevent *H. influenzae* from taking *N. gonorrhoeae* DNA and vice versa (Sisco and Smith 1979; Graves et al. 1982)]. However, to our current knowledge, DUESs are limited to some species of the Pasteurellaceae and Neisseriaceae families (Sisco and Smith 1979; Graves et al. 1982; Bakkali et al. 2004; Redfield et al. 2006; Bakkali 2007). They are not species specific, hence unable to prevent interspecific DNA uptake between species as distant as *P. multocida*, *H. influenzae*, *Actinobacillus actinomycetemcomitans* and *Mannheimia succiniciproducens*—these and other species have the same DUES (Bakkali et al. 2004; Redfield et al. 2006). Furthermore, it is argued that the extracellular DNA derives from dead cells and is therefore more likely to reduce fitness than confer advantage (Redfield 1988; Redfield et al. 1997). It is hard to see the point of bacteria taking up DNA for recombination to face challenging conditions if the DNA comes from dead cells that could not cope with those conditions (consider antibiotic treatment situations). One may argue that some of the extracellular DNA does not come from dead cells but is secreted by living ones or comes from cells actively killed by the competent bacteria. But DNA secretion and/or cell killing does not seem to be general characteristics of all competent bacteria, and the secreted DNA, or DNA from killed cells, is not as abundant as the one from dead cells. In addition, recombination of the strong (the killer) bacterium with DNA from the weak (the killed) bacterium is selectively counterproductive, and, for the secreted DNA to be of any selective use, ‘generous’ bacteria must ‘kindly’ secrete DNA that contains the advantage-conferring gene/mutation, so that others bacteria can take it up and use it to survive multiply and compete. This must be one of the utmost altruistic group selection situations! Another situation where extracellular DNA is not primarily from dead cells is biofilms. However, there, the DNA plays more a role of an aggregating matrix rather than a source of information. In addition, as with the chromosome repair hypothesis, I do not see why bacteria should degrade most of the DNA they bind to if what they want is recombination—why waste DNA and energy? Also, bacteria have no mate choice or sexual selection, and while unlikely to integrate non-homologous DNA, most competent bacteria take up DNA indiscriminately [see (Lorenz and Wackernagel 1994; Dubnau 1999)], even from eukaryotes! [e.g. *Acinetobacter* sp., *P. stutzeri* and *Ralstonia solanacearum* take up plant DNA (Bertolla et al. 2000; de Vries et al. 2001)]. What about the cost of taking up free bacteriophage DNA? And the ‘suicide plasmid’ (Li et al. 2001)? Competence allows uptake of DNA from dead cells, even of other species, it can thus hardly be seen as bacterial sex.

The *Competence for nutrition* hypothesis (Redfield 1993b) suggests that bacteria take up DNA for its nucleotides and has been adopted by different works [e.g. (Macfadyen et al. 1996; MacFadyen et al. 2001; Palchevskiy and Finkel 2006)]. Competence induction by starving *H. influenzae* (Herriott et al. 1970) and its reversion by adding nucleic acids precursors to the medium (MacFadyen et al. 2001) support this hypothesis. But, why should some species, including *H. influenzae*, ‘prefer’ con-specific DNA (Sisco and Smith 1979; Graves et al. 1982)? Why do they ignore DNA of other species? In fact, this ‘diet’ restriction discounts a ‘safer’ DNA, as con-specific DNA is more likely to recombine with the chromosome, while it could be damaged and/or coming from dead (i.e. unfit and aged) cells. Also, why should bacteria discard one DNA strand (i.e. 50 % of the potentially nutritive nucleotides)? Why does competence develop even in complex media [e.g. *A. calcoaceticus*, *N. meningitidis* and *N. gonorrhoeae* (Catlin 1960; Biswas et al. 1977; Palmen et al. 1993)]? Furthermore, *A. calcoaceticus*, *B. subtilis*, *N. gonorrhoeae* and *S. pneumoniae* release DNA to the medium while being competent (Lorenz et al. 1991; Moscoso and Claverys 2004; Hamilton et al. 2005). Similarly, anaerobic and heat shifts induce competence in *B. subtilis*, *S. pneumoniae* and even *H. influenzae* (Goodgal and Herriott 1961; Espinosa et al. 1980; Chapuy-Regaud et al. 2001) could stressed bacteria become hungry because of the stress? Competence involves DNA uptake from dead conspecifics and discharge of >50 % of the DNA bound. If it is a nutritional mechanism, it must be rather abnormal and evoking nutritional anomalies—namely cannibalism and bulimia.

Recently, competence was suggested to be sustained in *B. subtilis* by *episodic selection in favor of non-growing cells* in conditions where dividing cells are killed (Johnsen et al. 2009). At first, maintenance based on episodic situations where competence may confer advantage seems a priori a sensible possibility that may potentially work for whatever function competence may have. However, Johnsen et al.’s work (Johnsen et al. 2009) is based on *B. subtilis*, which is only occasionally (episodically) competent. Furthermore, when this species is competent, only ~20 % of its cells take up DNA (the environmental strains are even less competent than that [see (Young et al. 1963; Nijland et al. 2010) and references therein]. It is still to be seen whether episodic selection could apply to other species—especially the ones constitutively competent and those where ~100 % of the cells take up DNA. Similarly, this hypothesis is still to be tested with selective forces other than strong selections against dividing cells. Indeed, competence inducers such as heat, nutritional and oxidative stresses are expected to affect the cells ability to divide but would not kill the dividing cells. In any case, Johnsen et al.’s work (Johnsen et al. 2009) relies on deterministic modeling supplemented by tightly controlled (see unrealistic) experiments and even for the species tested (*B. subtilis*) the selective advantage it defends seems more in favor of non-growth in anti-growth conditions than of DNA uptake. The latter is suggested to enhance the advantage of non-growth (hence, the episodic selection hypothesis is a recombinational one), but the authors do not take into account that the DNA taken up from the environment could also be neutral, deleterious or parasitic (bacteriophage). They assume that the DNA taken up is always positively selected (80 % fitness increase) and abundant (for each cell, there is a good DNA molecule)—a clearly unrealistic situation. If a bacterium stops dividing in an antibiotic-containing environment, it has a chance to resist and survive and the last thing it should do is to gamble and take up DNA from that environment as most likely that DNA is from a dead antibiotic sensitive cell.

In my opinion, for competence to be a nutritional or a recombination/sex mechanism, at least two conditions must be met: (1) other cross-membrane mechanisms for metabolites transport or HGT must be insufficient for conferring adequate food supply or genetic diversification to bacteria (otherwise competence will not be needed and selection would not favor it), and (2) DNA uptake must not be accidental. In minimal media, competence may, at first, seem needed to acquire nucleotides from DNA. However, *B. subtilis* and *S. pneumoniae* develop competence although they secrete non-specific DNases that should degrade extracellular DNA and make its nucleotides available for cross-membrane transport (Burke 1982; Moscoso and Claverys 2004). As to genetic diversification, although competence seems ancestral and commoner than we currently know, it is less frequently reported than conjugation and transduction; meaning that its contribution to HGT might be lower. Currently, no single hypothesis can by itself explain a function for competence, nor how natural selection can favor it, without generalization of findings that might be specific to a single or group of species. As if we believed that every natural phenomenon needs to have a function for it to exist, we strive to pinpoint one for competence. Surprisingly, the subjacently assumed non-accidental nature of DNA uptake was never challenged. Understanding why bacteria bind and take up DNA in the first place could therefore be helpful.

DNA uptake involves type IV pili (T4P) [e.g. (Rudel et al. 1995a, b; Wolfgang et al. 1998; Chen and Dubnau 2003; Averhoff 2004; Varga et al. 2006)] and the less characterized but related structures called ‘pseudo-pili’ (Chen and Dubnau 2003; Chen et al. 2005). The well-characterized T4P, on which I will focus, are adhesion and motility structures that function by (1) polymerization of pilin subunits [pilA (Wu and Kaiser 1997), pilE in *Neisseria* (Fyfe et al. 1995)] assisted by the ATPase pilB [pilF in *Neisseria* (Lauer et al. 1993)], (2) secretion of the polymerized rod through the secretin pilQ (Collins et al. 2001), (3) adhesion of the T4P to a surface—normally via its tip, pilC (Rudel et al. 1995a, b; Kirchner and Meyer 2005)—and (4) its retraction by depolymerization involving the ATPase pilT (Merz et al. 2000). This sequence of events allows adhesion to surfaces, including host tissues [e.g. (Kubiet et al. 2000; Kirchner and Meyer 2005)], and its repetition using different T4P, normally located at alternate cell poles [e.g. (Mignot et al. 2005, 2007; Margolin 2006)], allows movement [see (Mattick 2002; Nudleman and Kaiser 2004; Christie et al. 2005)].

T4P is ~3–4 μm long and ~60 Å wide and has no central canal (Forest and Tainer 1997) (Fig. 1); so DNA cannot pass through it, and to enter the cell, it needs to cross the membranes through the ~6.5 ηm diameter pilQ pore (Collins et al. 2001). An agreed-upon mechanism describing DNA uptake was suggested in (Dubnau 1999; Chen and Dubnau 2003, 2004; Chen et al. 2005). But why and how do bacteria bind DNA in the first place? Although never directly stated, from the different hypotheses on the function and maintenance of competence, one can infer that there seems to be a *de facto* assumption that bacteria may actively seek DNA. But there is no evidence or indication to support such possibility. In fact, there may be evidence to supporting the more logical passive DNA binding possibility as there is at least one bacterium, *A. vinelandii*, that seems to bind DNA even when not competent (Doran and Page 1983). Therefore, the null (by-default) hypothesis (i.e. bacteria bind DNA just by ‘accident’) might still stand. In such case, with T4P being adhesion and motility structures, DNA uptake may as well be a by-product of these motility and adhesion functions.

**Fig. 1:**
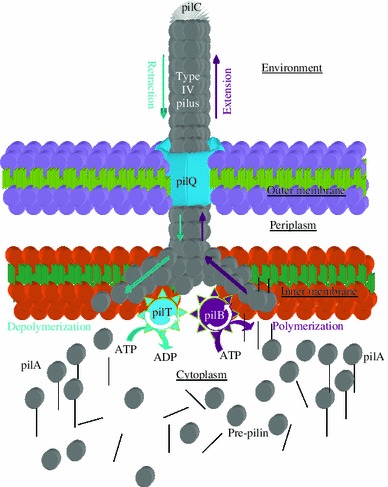
Simplified schematic representation of the structure of a type IV pilus

Obeying the laws of physics, bacteria move twitchingly if they adhere to surfaces that have higher inertia than the bacterial cell itself (Fig. 2). However, if T4P adheres to a DNA fragment that is smaller and has less mass and electrostatic energy than the bacterial cell, its retraction should pull the DNA toward the cell rather than the cell toward the DNA (Fig. 2). Once at the pilQ pore, the DNA [~2 ηm diameter and ~50 ηm bend-persistence length (Manning 2006)] would kink and form a loop ~43.5 ηm wider than the pore. The T4P and the pore would thus be blocked. This resembles an imaginary 12 cm round arm pulling a 4 cm diameter 1 m bend-persistence length tube through a 13 cm diameter window. Obstruction of the T4P and/or the membrane pore could be a selecting force for presence of the membrane-localized nucleases that reduce DNA stiffness by eliminating one of its strands [e.g. endA, nucA and nucT (Provvedi et al. 2001; Berge et al. 2002; O’Rourke et al. 2004)]. While the nuclease is located at the only membrane, Gram-positive bacteria have and they necessarily degrade and release the DNA strand into the medium; for Gram-negative bacteria, only inner membrane nucleases are thus far experimentally detected (and it releases the degraded DNA into the periplasm). Outer membrane nucleases in Gram-negative bacteria are thus far just a theoretical possibility that is neither experimentally proven nor excluded. Still, whether the impasse happens at the inner or at the outer/only membrane, the outcome is the same; a blocked pilus and pore structures. Bacteria might hence be accidentally binding and taking up DNA as they try to move (e.g. when escaping stressful conditions [Kozlovsky et al. 1999; Lacasta et al. 1999; Tang et al. 2004; Nachin et al. 2005; Wang et al. 2006; Gomez-Gomez et al. 2007)] (Fig. 3). Indeed, bacterial pili are known to bind and introduce ‘unwanted things’ into the cell (e.g. plasmids and bacteriophage DNA [Bradley 1975; Karaolis et al. 1999)], so DNA binding and uptake during competence could be just another accidental by-product of the pili extension and retraction. One could thus *speculate* that bacteria not known as competent could be competent less frequently or under strict conditions, might not use T4P or similar structures for adhesion and twitching motility, their adhesins’ specificity might exclude DNA or might have membrane-localized, periplasmic and/or cytoplasmic 5′-3′ and 3′-5′ nucleases that degrade any DNA bound or taken up—so that the DNA end up with no possibility of entering the cell and/or surviving in the protoplasm to recombine with the chromosome and, thus, transform the bacterium. Indeed, the reducing effect of the nucleases on bacteria′s transformation by DNA uptake capacity is demonstrated by the increase in transformability of the cyanobacterium *Synechocystis* sp. after elimination of its cytoplasmic exonuclease, RecJ (Kufryk et al. 2002).

**Fig. 2:**
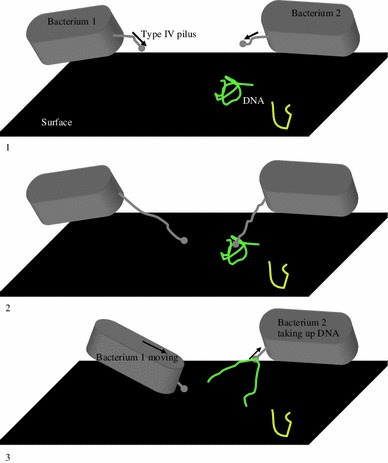
Bacterial twitching motility and the possibly unintentional binding and uptake of DNA*. Arrows mark* the moving part and its direction

**Fig. 3:**
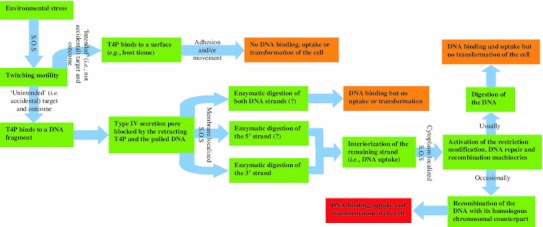
Schematic explanation of the accidental nature of DNA binding by type IV pili of the motile/adhering bacteria and the possible outcomes with regard to DNA uptake and bacterial transformability. The interrogation marks signal options that while possible remain speculative at this point as they are still not experimentally demonstrated

When facing unfavorable conditions, bacteria would be stressed and could increase their motility [e.g. (Kozlovsky et al. 1999; Lacasta et al. 1999; Jelsbak and Sogaard-Andersen 2000; Tang et al. 2004; Nachin et al. 2005; Wang et al. 2006; Gomez-Gomez et al. 2007)]; meaning that T4P extension and retraction cycles increase and so should be the likelihood of DNA binding and uptake. Accordingly, T4P respond to factors like quorum sensing (Kendall and Sperandio 2007), pH (Brener and DeVoe 1983; Manetti et al. 2010), and temperature (Liles et al. 1998), and T4P-dependent motility is light regulated and linked to transformation in *Synechocystis* (Bhaya et al. 2000; Yoshihara et al. 2002). Indeed, competence was suspected to be a general SOS stress response (Li et al. 2002; Claverys et al. 2006; Charpentier et al. 2011). It is even deadly to a sub-fraction of *S. pneumoniae* (Steinmoen et al. 2002)—probably because this species produces a competence-stimulating peptide that is also antibacterial (Oggioni et al. 2004). Antibiotics misuse—which does not always eliminate the target while stressing non-targeted bacteria—might therefore not only be selecting for resistant strains, but also increasing DNA uptake and the likelihood of acquiring drug resistance and virulence. This seems true at least for *S. pneumoniae* and *L. pneumophila* where antibiotics treatment induces competence (Prudhomme et al. 2006; Charpentier et al. 2011).

The hypothesis suggested here tackles the weaknesses of the previous ones and explains experimental observations including (1) instead of taking full advantage of the DNA, bacteria get rid of as much of it as they probably can (they therefore seem not to want it), (2) *A. vinelandii* seem to bind DNA even when not competent (Doran and Page 1983) (a clear case of accidental DNA binding), (3) extracellular DNA is important for biofilm formation (Whitchurch et al. 2002) (the case of biofilms could be relevant for the current hypothesis as it shows that bacteria can stick to DNA not only for the purpose of taking it up. Hence, at least in this occasion, DNA seems an adhesion substrate rather than a genetic information or nutritional material), (4) DNA is a major constituent of the neutrophil extracellular bacteria traps (Brinkmann et al. 2004) (that probably exploit the accidental binding of DNA by bacteria to entrap and phagocyte them), and (5) all competence inducers cause stress (e.g. starvation, heat, antibiotics) that increases twitching motility and, thus, pili activity (polymerization and retraction) hence the consequent possibility of DNA binding, uptake, and the following degradation or occasional integration into the chromosome [e.g. (Kozlovsky et al. 1999; Lacasta et al. 1999; Jelsbak and Sogaard-Andersen 2000; Tang et al. 2004; Nachin et al. 2005; Wang et al. 2006; Gomez-Gomez et al. 2007)]. (6) Transformation and adhesion are linked in *H. pylori* (Lin et al. 2006).

The inhibition of competence in *H. influenzae* by adding nucleotide precursors to the medium (Macfadyen et al. 1996, 2001), the phase variation in DNA uptake in some species, the up-regulation of several cytoplasmic proteins—especially nucleases and DNA repair ones—and the overrepresentation of DUESs in the genomes of some species with preferential uptake of con-specific DNA (Sisco and Smith 1979; Graves et al. 1982; Bakkali et al. 2004) seem to contradict the hypothesis suggested here. However, addition of nucleotide precursors relieves the starvation stress that induces competence in *H. influenzae*, which may reduce cell motility and accidental DNA binding and uptake. On the other hand, phase variation in DNA uptake is due to variation in T4P production and states of adhesion and motility [e.g. (Villar et al. 2001)]. Similarly, nucleases, DNA repair proteins and other membrane-localized and cytoplasmic proteins are not exclusive to the competence state [e.g. EndA (Claverys et al. 2006) and SsbB (Thanassi et al. 2002)] or to competent species [e.g. DprA (Smeets et al. 2006) and RecA (Sung and Klein 2006)]. They may, thus, not have evolved as competence proteins and might be better seen as SOS response systems for dealing with DNA binding or interiorization into the cell as part of their role in secretion, transport, enzymatic, chromosome repair and/or defense against ‘parasitic’ DNA systems. On the other hand, proteins such as *B. subtilis* ComEC and ComEA have thus far no other demonstrated function but in competence. However, these proteins were detected in only few species and are not fully studied as to discard their involvement in other processes. In fact, at least ComEC is orthologous to *H. influenzae* Rec2, which is also involved in phage recombination (Kupfer and McCarthy 1992), *S. pneumoniae* CelB, which is also involved both in biofilm formation and virulence (Wu et al. 2012) and in the use of and growth on cellobiose (Zeng and Burne 2009; Shafeeq et al. 2011), and to *N. gonorrhoeae* ComA gene, which is known to be involved in pilin variation (Facius and Meyer 1993). All in all, while the membrane-localized proteins (such as the Com and Nup proteins are constitutive parts of these structures, the cytoplasmic ones [for a review see (Claverys et al. 2009)] would be up-regulated upon detection of foreign and damaged DNA in the cytoplasm no matter what its origin could be, even phage DNA and DNA artificially introduced by electroporation. Once the DNA has been accidentally bound, the cell uses different proteins to deal with the situation. This would explain why in *B. subtilis* entry of exogenous DNA occurs at the cell poles (Hahn et al. 2005; Chen et al. 2006; Hahn et al. 2009), where the pseudo-pili as well as the competence-related proteins are located (Kaiser 1979; Henrichsen 1983; Kaiser 2000; Mattick 2002; Hahn et al. 2005, 2009), and where the polymerization–depolymerization cycles of the pili allow motility (McBride 2001; Mignot et al. 2005). Some of the competence-related proteins (such as the Com proteins) are expressed even by non-competent bacteria [such as *Streptococcus sanguinis* strain 133–179 (Zhu et al. 2011)]. As to DUESs, they are hitherto limited to some Pasteurellaceae and Neisseriaceae species. Their evolution seems therefore subsequent to that of competence, so their evolution must be related to specificities in the DNA binding by these species. Given that T4P, especially of pathogens, show differences in their adhesion specificities [e.g. (Kubiet et al. 2000; Kirchner and Meyer 2005)], DUESs may have been gradually driven to overrepresentation in unconstrained regions of these species’ genomes (all pathogenic) as a possible consequence of this adhesion specificity [Bakkali in preparation]. This would inevitably result in preferential uptake of con-specific DNA. This dynamic is what the molecular drive hypothesis on DUESs evolution suggests [see (Bakkali et al. 2004; Bakkali 2007)]. At the end, the accidental binding and uptake of DNA nicely fits the suggested model on DUES evolution (Bakkali et al. 2004; Bakkali 2007), as well as the facts that DUESs must have evolved after competence evolution, they are limited to pathogens, the latter have specific adhesion to their host tissues, and this specificity relates to characteristics of the T4P (which binds the DNA).

In conclusion, DNA uptake could be an ‘unintended’ by-product of bacterial adhesion and twitching motility. However, accidental binding and uptake of DNA does not *completely* rule out any of the available hypotheses on the potential benefit of competence, and cells may *occasionally* benefit from *different* contributions of competence *depending on the species and/or situation*. For instance, bacteria with indiscriminate DNA uptake may benefit from potential occasional nutritional contribution of DNA uptake (if DNA uptake coincides with a nucleotide starvation situation), and an occasional advantage of recombination may help species ‘preferring’ con-specific DNA and those frequently exposed to antibiotics/stress (if DNA uptake coincides with a challenging environment and the DNA taken up happens to be an advantageous piece from a cell that died for reasons unrelated to the challenging situation). However, the coincidence between DNA uptake and the cell’s need for nucleotides is not always guaranteed and may not be as frequent as to explain the evolution and maintenance of competence. Less likely is the coincidence between uptake of an advantageous DNA and the cell’s need for that particular piece of DNA. Hence, competence could also be selectively neutral and drifting throughout the bacterial evolutionary history. It may have been lost in some bacteria while still drifting in others. This could explain its sporadic distribution across taxa. Whatever the case, antibiotics misuse is most likely favoring the increase and spread of DNA uptake and competence [e.g. (Prudhomme et al. 2006)] while selecting for bacteria capable of acquiring new genetic material that potentially confers drug resistance. The consequent increase in multidrug resistance and virulence adds to increased bacterial dispersal assisted by extensive worldwide displacement of humans, livestock and other goods.

Finally, it is worth mentioning that, if demonstrating the existence of existing things is hard, demonstrating the non-existence of non-existing things is much harder. Some of the crucial tests on the validity of my hypothesis include examining whether bacteria sense the presence of extracellular DNA, testing whether bacteria actively search for DNA and analyzing DNA binding to cells in the presence of competing non-nutritive substances that are devoid of genetic information.
